# Engaging with discomfort when learning sexual and reproductive health and rights. A qualitative content analysis of public health students’ experiences of active learning in an international master’s level course

**DOI:** 10.1186/s12889-026-29680-4

**Published:** 2026-09-29

**Authors:** Camilla Palm, Karin Båge, Magnus Hillman, Argyro Pavlidou, Tobias Herder

**Affiliations:** 1 Centre for Sexology and Sexuality Studies, Department of Social Work, Faculty of Health and Society, Malmö University, Malmö, Sweden; 2 Department of Global Public Health, Karolinska Institutet, Stockholm, Sweden; 3 Center for Teaching and Learning, Faculty of Medicine, Lund University, Lund, Sweden; 4 Social Medicine and Global Health, Department of Clinical Sciences, Faculty of Medicine, Lund University, Malmö, Sweden; 5 IHE – The Swedish Institute for Health Economics, Lund, Sweden

**Keywords:** Sexual and Reproductive Health and Rights, SRHR, University education, Learning environment, Qualitative content analysis

## Abstract

**Background:**

Within most academic sexual and reproductive health and rights (SRHR) curricula, topics considered sensitive within the broader social context are frequently addressed. To gain a better understanding of how active learning can be promoted in education about potentially sensitive topics, this study aimed to explore the experiences of active learning among an international student cohort attending a master’s level course in SRHR.

**Method:**

This qualitative study draws from 11 in-depth interviews with students previously enrolled in a master’s level course in SRHR at the Faculty of Medicine, Lund University, Sweden. Interviews were analyzed using qualitative content analysis.

**Results:**

The analysis resulted in two sub-themes; Co-creating a safe environment for active learning in SRHR and Managing challenges and catching opportunities for active learning in SRHR, and one overarching theme; Considering self and others when engaging with uncomfortable dimensions of learning SRHR.

**Conclusion:**

The findings indicate that discomfort has the potential to be productive for active learning. However, this necessitates that the learning environment is intentionally curated by both facilitators and students into a learning space characterized by trust.

**Supplementary Information:**

The online version contains supplementary material available at https://doi.org/10.1186/s12889-026-29680-4.

## Key message

**What is already known on this topic**

Learning about sexual and reproductive health and rights (SRHR) often involves engaging with potentially sensitive topics that can generate discomfort and influence active learning. Research on discomfort and pedagogical strategies mainly comes from facilitators’ experiences, while learners’ perspectives are understudied.

**What this study adds**

This study conceptualizes forms of discomfort in SRHR education and shows how it contributes differently to individual and collective learning or become ethically and socially costly.

**How this study might affect research**,** practice or policy**

The findings offer pedagogical insights for educators by identifying teaching strategies that foster trust and relational safety, enabling discomfort to function as a recourse rather than a barrier to active learning in SRHR education across contexts.

**Supplementary Information:**

The online version contains supplementary material available at https://doi.org/10.1186/s12889-026-29680-4.

## Introduction

Teaching topics that may be considered sensitive in higher education presents both pedagogical opportunities and challenges. Across disciplines such as criminology, social work, public health and sexuality studies, teachers and students are confronted with value-laden, emotionally charged, and potentially distressing themes [1–3]. Such topics have variously been defined as sensitive topics or controversial issues [4]. Here, we use the term sensitive topic to denote issues that may evoke strong emotions, conflict, or are perceived as sensitive by students. In literature on teaching controversial issues, increasing attention has been paid to how classrooms can foster democratic skills such as dialogue and critical thinking, while also highlighting pedagogical dilemmas when strong emotions and divergent opinions emerge [5, 6].

Within most academic sexual and reproductive health and rights (SRHR) curricula, topics considered sensitive or taboo within the broader social context are frequently addressed, such as abortion, comprehensive sexuality education, and diversity in sexual orientation. In addition, SRHR courses designed to increase awareness of power relations and expose students to diverse perspectives, have been reported to be challenging and may evoke strong feelings or resistance [4, 7]. Davis [1] argues that, unlike many other sensitive issues, sexuality and the body are challenging to teach and learn because they are simultaneously taboo in public discourse and also tied to the personal; students’ and teachers own bodies, identities, and relationships. As such, everyone present in the classroom has personal experience of the topic, but few reference points beyond their own. Therefore, students often expect learning to be about the body and sexuality “as they know it” [1]. SRHR courses thus constitute arenas where norms and values related to sexuality, the body, and health are exchanged and negotiated, and both teachers and students enter the classroom with their own ideas, experiences, and assumptions [1]. Hereafter we use the term *facilitators* to reflect the role teachers play when engaging students in active learning activities.

### Student participation and discomfort

Active learning is commonly defined as instructional approaches that engage students in the learning process by requiring them to participate in meaningful learning activities and reflect on what they are doing, rather than be passive recipients of information through lectures [8]. In this study, active participation in team and classroom discussions is understood as one important activity through which active learning may occur [9]. Promoting student engagement and collaborative learning environments has been shown to support deep learning, knowledge retention, and critical thinking more effectively than passive instruction [8, 10]. Therefore, active learning may contribute to intended learning outcomes such as independent and critical thinking skills, as emphasized for example in the Swedish Higher Education Ordinance [11]. In SRHR courses, such skills may entail developing perspective-taking, including awareness of one’s own beliefs and values, critical examination of their social and cultural foundations, and recognition of how one’s own and others’ perspectives are informed by specific historical and sociopolitical contexts [see for example: 1, 12, 13]. Learning that contributes to such shifts in perspectives, and to the critical self-examination of social norms and values has been described as transformative learning [14]. In the context of SRHR, traditional education may, for example, involve students learning about laws regulating same-sex sexualities in different countries. Transformative learning, however, involves developing an understanding of, and critical reflection on, the social norms that shape one’s own and others’ attitudes as well as the laws. As such, active learning can contribute to a deeper understanding of complex SRHR issues that are both personal and political, and span both health and human rights, and a necessary competence for all professionals involved in SRHR work.

Nonetheless, active learning may be challenging to implement [15]. Activities such as classroom discussions, which can help students develop perspective-taking skills and conceptual understanding [16], can be pedagogically challenging when the topic is considered sensitive by students or facilitators. While sensitive topics can facilitate dialogue, reflection, and transformative learning [17], they can also provoke discomfort, silence, or student withdrawal [18]. Students may avoid engagement due to fear of being stereotyped [19], perceived misalignment with the facilitator or majority opinion [20], or concern about potential conflict or regret over disclosing personal views or experiences [21]. Sanchez et al. [7] found that students may respond emotionally to topics if they perceive the class as unfriendly and non-inclusive. Research indicates that classroom culture and facilitators’ approach are essential for fostering dialogue; authoritative questioning or overly didactic styles may reduce participation in discussions [22]. In their quantitative study, Cebula et al. [23] suggest that interpersonal aspects and facilitator attitudes such as warmth and ability to create a safe learning environment, are more important than specific pedagogical techniques.

While much literature has engaged with strategies for teaching sensitive topics, much of the literature is based on facilitators’ perspectives and experiences, often using an autoethnographic approach [see for example: [24–26]]. Student perspectives in higher education remain less explored [23]. This study addresses this gap by examining university students’ experiences.

To gain a better understanding of how active learning can be promoted in education about potentially sensitive topics, this study aimed to explore the experiences of active learning among an international student cohort attending a master’s level course in SRHR. While SRHR provides the empirical focus, we expect the findings to inform teaching on sensitive topics across disciplines, including political science, human rights, social work, criminology, medicine, and all health-related sciences, as well as beyond higher education contexts.

## Methods

### The context: an international higher education course in SRHR in Sweden

The current study explored experiences among students attending a five-week campus course (7.5 credits) on SRHR in the international Master’s Program in Public Health at Lund University (LU), Sweden. Annually, the program enrolls around 40 international students from diverse academic disciplines and countries of origin. The SRHR course is based on active learning activities that require students to engage in constructing knowledge through interaction with peers, facilitators, course material and study visits, i.e. through discussion, collaboration and problem-solving [8]. It covers potentially sensitive topics, including abortion, transactional sex and female genital mutilation or cutting (FGM/C). To prepare students to engage with these topics and others, introductory learning activities focus on establishing joint so called ground rules for a constructive learning environment [6] and encourage reflection on prior understandings of SRHR and how these have been shaped. The course also distinguishes between private and professional approaches to discussions, where sharing private experiences is discouraged, and intentionally, acknowledges the challenges of active learning in discussions on potentially sensitive topics.

### Data collection

Data were collected through in-depth interviews with master’s students who had attended a 7.5-credit course in SRHR at the Faculty of Medicine, LU. A semi-structured interview guide was developed, covering themes such as *topics considered sensitive*, *facilitators’ and students’ influence on learning environment*, and *motivators and barriers to active participation.* A pilot interview was conducted in November 2024. As no substantial revisions were required, the interview was included in the analysis. All 41 students who had attended the course during the fall semester of 2024 were invited to participate through individual emails and a classroom announcement after course completion. Eligible participants contacted the interviewers (CP and KB) directly to book an interview. Sampling was continued until sufficient information power was achieved. Information power depends on elements such as the breadth of the study aim, sample specificity, and the quality of the collected data. Richer data, a more narrow aim, and a specific sample of study participants require fewer participants for the intended analysis [27]. Interviews were conducted by CP and KB. All students who expressed interest in participating were offered an interview. Prior to the interviews, participants received written information and opportunities to ask questions about the study. Verbal informed consent was provided and documented via audio recording. Interviews were conducted in English on Zoom, and were audio recorded. Recordings were transferred to a secure data management and analysis platform at LU, where they were transcribed verbatim and pseudonymized through the removal of identifiable information, such as names, countries, and educational backgrounds, when mentioned. In the article, participants are referred to as P1-P11.

### Participants

Eleven interviews were conducted between November 29th 2024, and April 4th 2025, lasting 39–78 min (Mean: 61 min). Participants reflected the international student cohort and included students from Europe (*n* = 7), the North Americas (*n* = 2), and Asia (*n* = 2). The median age was 26 years (Mean: 28 years). Ten identified as female and one as male.

### Analysis

**Fig. 1:**
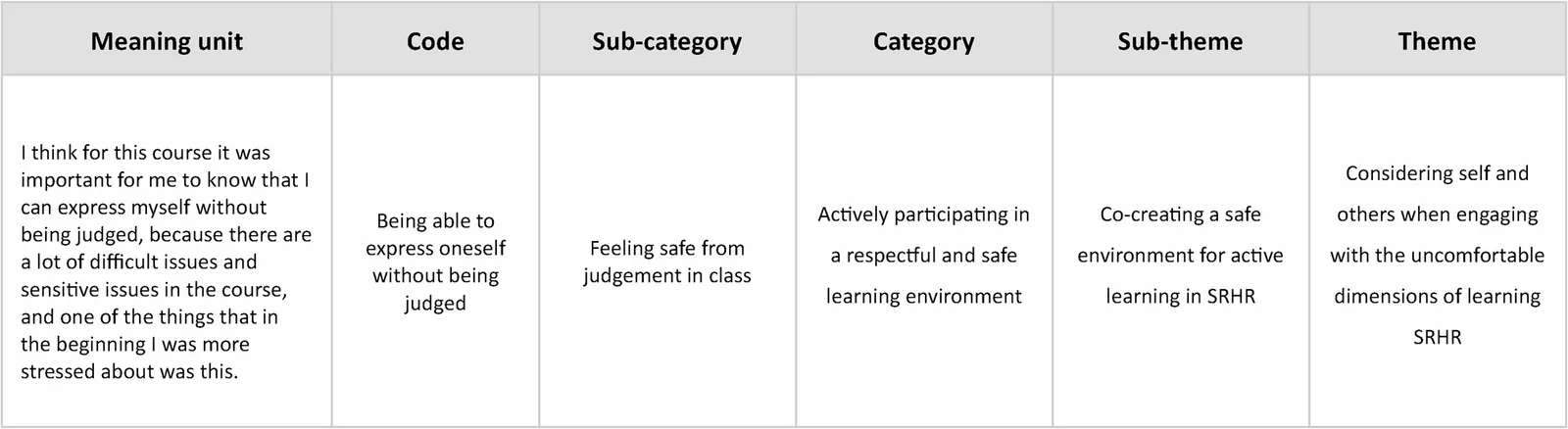
Example of the analytical process

A qualitative content analysis was conducted to explore students’ experiences [28], departing slightly from the process as described by Granheim and Lundman by not condensing the meaning units identified. The analysis took an inductive approach [29]. It started by identifying and coding meaning units of the transcripts directly, sorting the codes into manifest sub-categories and categories, then identifying the sub-themes and an overarching theme representing the latent, underlying meaning of the student experiences [30]. The analysis was led by the project lead TH, with coding carried out by KB and TH using OpenCode 4.0. First, KB and TH coded one interview each whereafter codes were compared and discussed to reach an agreement on the coding structure. Then, TH and CP identified categories and themes. The preliminary results were discussed among all co-authors, and a member check was carried out, before finalizing the analysis. An example describing the analytical process is illustrated in Fig. 1.

### Reflexivity statement

The research team behind this study have varying involvement in the course under study. TH was the course leader and has developed and led the course since 2019, together with colleagues from LU. KB and CP both give guest lectures in the course. AP is a former student of the course, who was involved as a research assistant in the project. MH is an educational developer at the Faculty of Medicine, LU, and not involved in the course. This diversity provided multiple perspectives but also introduced potential biases related to familiarity with the course content and teaching methods. To mitigate these, the team engaged in regular discussions to critically reflect on their roles, assumptions, and interpretations throughout the research process.

## Results

**Fig. 2:**
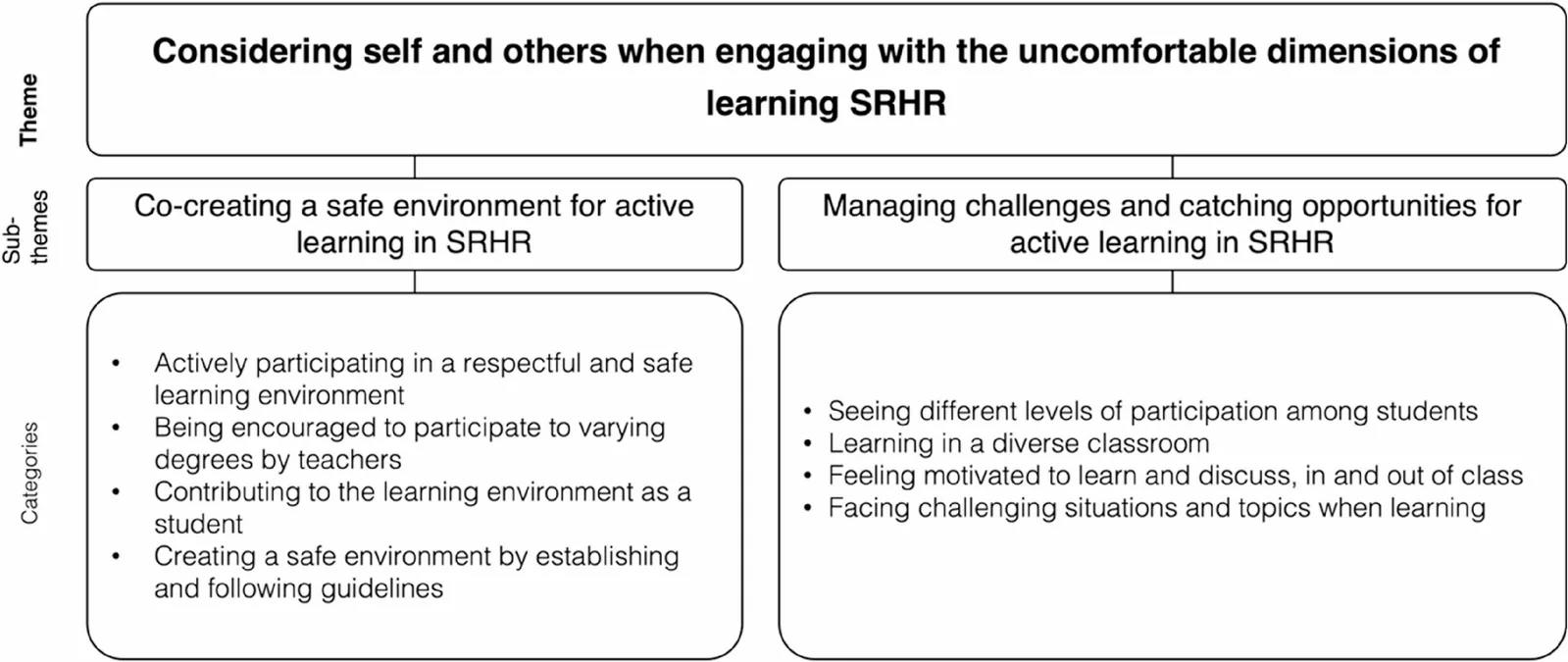
Overview of the main results: A latent theme of meaning, two descriptive Sub-themes, and eight manifest Categories

The analysis resulted in 1159 codes sorted into 39 sub-categories. These sub-categories were further sorted into eight Categories, two sub-themes and one overarching theme; *Considering self and others when engaging with the uncomfortable dimensions of learning SRHR.* An overview of the results is illustrated in Fig. 2.

### Sub-theme 1: Co-creating a safe environment for active learning in SRHR

Participants described that feeling safe, having trust in facilitators and peers, and feeling free from fear of judgement, facilitated their active engagement in learning activities.

I think for this course it was important for me to know that I can express myself without being judged, because there are a lot of difficult issues and sensitive issues in the course, and one of the things that in the beginning I was more stressed about was this. So I think it’s a friendly environment and that you feel that you are respected from the teacher but also from the other students. (P1)

Here, students reported increased comfort with active participation in learning activities as the course progressed. Students ascribed this increased comfort to both facilitator strategies and peer behavior. While the students acknowledged the facilitators’ intentional efforts to encourage active participation, it was not always clear to them how this was achieved.

I think in the SRHR course specifically everyone felt safe and had the opportunity to be engaged, it’s the way that the lessons were taught, I don’t know how the teachers managed to do it, but they did. (P3)

Many attributed their confidence in participating in learning activities to the mutual respect that was discussed at the beginning of the course.

SRHR was a very interesting subject for me, and not only because of the topics but because of the approaches the teachers used and…because we were talking about this… mutual respect, active participation and everything. (P2)

Another factor contributing to participation was the sense that facilitator-student relationship felt less hierarchical than in their earlier education. They expressed how facilitators actively invited students to discussions and, in some exercises, also encouraged students to question what the facilitator had said.

I would say that in Sweden I found it very interesting that in the lecture session, it’s very interactive. It’s kind of communication between teachers and student and teacher makes their lecture in a way so that everyone can participate and share their view. (P11)

Where I am coming from, the education system is […] it’s like the teacher is standing in front of you […] it’s like you need to have this fearful respect for them (P9).

The introductory learning activities were seen as supporting students’ ability to actively participate by encouraging discussions in ways they perceived as “academic” rather than personal.

During the SRHR course the teachers always emphasize on respect and avoiding personal experience stuff, I think it is very nice /…/ to keep the discussion in an academic way, and make sure this is serious and respectful environment to talk about everything. (P10)

I think that if the teacher did not mention that, that everyone would not have thought about that [not sharing personal experiences in class]. So, it is really important that this introduction happens in the beginning, that everyone is on the same page. (P3)

In the introduction, it was acknowledged that certain topics could evoke emotions, and students were given the option not to participate in learning activities or leave the class as needed. This option was described as important and perceived as contributing to their sense of safety, as it allowed them to manage their level of engagement with potentially emotionally demanding topics.

While active participation in discussions was seen as encouraged and sometimes required as part of the examination in assessed seminars, students also described having felt pressure to participate in discussions. One example included having been asked about specific policies in one’s home country.

It feels like every time abortion comes up they’re like ‘what’s going on over there.’ It kind of puts me on the spot, which I don’t mind because it’s not like that’s super personal to me, but if I had experiences, if I had negative experiences /…/ then it would make me feel very different being put on the spot to talk about abortion policies in [my country]. (P5)

Even if ground rules were established at the beginning of the course, students expressed having been concerned that guest lectures were not aware of the guidelines. Trust developed over time with the main facilitators, while guest-led sessions did not always provide the same sense of safety.

Another recurrent theme mentioned as important for the co-creation of a safe learning environment, was how facilitators modeled non-stigmatizing language use regarding sensitive topics. The interviewees described how it contributed to their comfort to participate in learning activities and discussions. Facilitators were not perceived as enforcing specific terminology but seen as offering alternatives. One example raised was the introduction of People First language [31], used in a learning activity related to stigma in HIV prevention.

Similarly to how facilitators’ use of language modeled specific ways of speaking, the facilitators’ active engagement in discussions and learning activities was perceived as modeling how to participate. Participants described facilitators as genuinely interested in students’ perspectives and experienced that facilitators provided alternative viewpoints when they joined student discussions.

You know the best feeling I can see or feel from the [teacher is] how they try to be…hrmmm… I’m going to say on our level […] sometimes I feel like they enter into the discussions like with us, it’s not like their, yes, of course they are leading the discussions, because I mean somebody needs to, but they can like add [for us to] consider also this. Like there is a word, “consider,” and I heard it more often in the course than somewhere else before. (P9)

While facilitators’ strategies were highlighted as important for active learning, students emphasized that their experiences were also shaped by a shared responsibility among students to maintain a respectful classroom environment.

I think that’s also part of the responsibility of every student. /…/ Of course, peers’ attitudes would be really important during the course and since the teacher already emphasized about respectful and the serious atmosphere that we need to keep during discussions, I’d say everybody do a pretty good job. At least I didn’t feel uncomfortable about the peers’ reactions… (P4).

Participants described how they felt discussions became less meaningful when students adopted a disrespectful or confrontational tone. One example involved a classroom discussion about terminology for people selling sex.

Their tone was charged so that then the conversation became a bit useless I thought. Because again it is not about being right or wrong, but it is just about understanding where the other position stands as well and then can coexist with your worldview without having to crush it. (P6)

Respect for others’ views was a recurring theme in the interviews. Exposure to different perspectives or debating opposing ideas was described as contributing to their own learning. Active listening, introduced at the start of the course, was also viewed as crucial for learning. At the same time, the desire to show respect for peers was also perceived as a barrier to participation, as students hesitated or avoided to share views for fear of offending others.

We had someone from an area with a high prevalence of genital cutting so that adds a level of like, okay, I want to be extra sensitive because this person is here and I don’t want to say anything that would specifically offend them, because I don’t want them to feel uncomfortable. (P5)

The diverse backgrounds of the students in the class contributed to this fear of offending others. Through the interviews, it became evident that the students recognized that both facilitators and students have a joint responsibility for creating a learning environment perceived as respectful and safe. They also saw themselves as active contributors to this process, a process that continued to develop during the course. Moreover, they emphasized the importance of feeling allowed to make mistakes and valued that facilitators explicitly encouraged that. Facilitators’ emphasis on developing knowledge about diverse perspectives, as well as students’ trust in facilitators’ ability to offer gentle corrections to factual errors, further contributed to this sense of safety. One student recounted how, after making a comment they felt was misunderstood by peers, feelings of fear and of being misinterpreted initially emerged. However, the ensuing dialogue, both within and outside the classroom, contributed to a sense of being accepted, even though full mutual understanding was not necessarily achieved. Students also engaged in the co-creation of a safe environment by regulating the intensity of discussions through tactics such as moving on to other topics when discussions began to feel too heated during student-led seminars. Others described an approach in which they assumed good intentions on the part of their peers. This process is captured by the first sub-theme; Co-creating a safe environment for active learning in SRHR.

### Sub-theme 2: Managing challenges and catching opportunities for active learning in SRHR

Interviewees perceived that participation was uneven among students in class, which they attributed to several factors. Those who described themselves as non-participators explained how they preferred to learn by observing and listening, especially when they felt they lacked in-depth understanding or knowledge of the topic being discussed.

When you are forty people from different countries, every time you hear someone you understand more about that person and you learn from [them], and I’m a person that prefers to listen and learn… because I think it’s stronger to learn, because I know my opinion, for me it’s better to hear thirty-nine other opinions and learn from them than take up space and speak all the time about myself or like what I think (P3).

The quote above illustrates students’ self-awareness of their level and type of participation. Equal participation was perceived as more challenging to achieve in a lecture compared to smaller group seminars. The quote also highlights the value students placed on learning within a diverse classroom. The students described how it contributed to learning by enabling exchange of different experiences and perspectives between peers. Yet, the diverse backgrounds of the class were also seen as contributing to an additional challenge in active participation, as language skills were seen as a barrier to participation.

I felt that I couldn’t express my opinion because my English is not or was not very good and that, uhm, maybe to express my ideas, would take more time and there are always other students who uh like lead the activities or lead the discussions, eh, so maybe a few times I just felt that [it] would be better just to [stay silent] and to listen (P8).

Learning in a diverse classroom made experiences related to culture and cultural dimensions of SRHR present in the learning environment. While this was perceived as something positive overall, and contributing to cultural awareness, it was also described as contributing to uncomfortable situations.

I think that the biggest difference here is that we are so diverse in terms of cultures and backgrounds so, sometimes it’s challenging and maybe it wasn’t the most comfortable form of activity or learning in this course but I believe it’s still important and we need to do such things as well, just to learn and to grow, to understand how to interact with people who are from different cultures, countries, for example, and from different backgrounds. So I feel like even if it wasn’t really… comfortable for me during this course it’s still a really good experience to have (P2).

Preconceptions about cultures were also present in the interviews, both in students’ reflections on how their own presumptions had been challenged, and in accounts of assumptions expressed by others. One example related to a discussion on FGM/C, when a student with personal experience strongly disagreed with another student’s statement about FGM/C. The situation caused discomfort and raised questions about the facilitator’s role. Students widely agreed that facilitators have a responsibility to intervene when discussions escalate or when statements risk reinforcing misconceptions. They argued that failure to address such statements may reproduce misinformation. Some felt facilitators did not always dare to correct students for fear of causing discomfort. Others appreciated how facilitators handled incorrect or insensitive statements by picking up constructive aspects of a student’s contribution while introducing additional perspectives, information, or context without explicitly correcting the student.

Among the interviewees, gender was also perceived to influence their active participation. A male student expressed hesitation to contribute to discussions on issues primarily affecting women, as he felt it was not his place to speak on experiences he did not share. Conversely, a female student described discomfort in discussions about SRHR topics in the presence of men, as it was her first experience of doing so.

This is what is new, and I feel awkward is because we…I am used to situations like the female standing together to face the female problem. And when there are males around, it feels like, I will worry about the males’ attitude, if they are joking around or say something really bad. /…/ but that didn’t happen during the course so I appreciate that, they are all showing respectful attitude toward that. (P10)

The interviewees highlighted several situations they experienced as challenging and uncomfortable, such as seeing images of genitals in class, engaging in discussions on cultural understandings of certain practices related to SRHR, or having their preconceived ideas questioned. When asked what topics they considered sensitive, interviewees mentioned FGM/C, abortion, sex work/transactional sex, sexual violence, and topics concerning LGBTQ, as particularly sensitive. However, despite discomfort, participants often described such challenges as valuable learning opportunities in themselves.

I also think it was good for me because if I hadn’t been - what do we say - challenged to take this course I would likely not have dived into it and [found] more information. /…/ I think it’s a very crucial part of learning, to get challenged like in one way or another. If it’s from the teacher or your fellow students or if it’s the topic. […] because it’s something that you maybe not […] would have chosen or maybe find a little bit uncomfortable, it can maybe change you in some way and make you learn some new things as well (P7).

Students described how learning and participation extended beyond the classroom. In many instances, classroom discussions had continued with peers, family members, partners, and roommates. Exposure to new perspectives had also motivated some to further learning, for example by seeking additional information to confirm or revisit their own views.

I think it even enhanced my learning about the subject because I uh… I did some more research actually to like prove my point, for myself, I didn’t like prove my point to the other people but for myself to validate that what I’m thinking is what I want to think and that there’s like research for it. (P4)

One student also emphasized the importance of discussing course content outside the classroom to engage with perspectives beyond those represented in the academic settings, arguing they do not necessarily reflect “people from the outside.”

I think just being in the university bubble […] and all of us kinda shaking hands be like, “abortion is a service”, like yes okay sure, but also how does the rest of the world actually see it? And it feels like it’s missing so much peoples’ opinions because we can’t see them, and we can’t see them because we just don’t give them voices. […] [It] is sometimes a little bit, it takes away from other conversations and other opinions of people that are from outside. (P6)

Learning in a culturally and academically heterogeneous classroom offered opportunities for students to learn from their peers, and to challenge and develop their own understanding of SRHR. Being in this learning environment involved facing uncomfortable situations that students had to navigate, but successfully doing so led to new learning opportunities emerging, captured in the second sub-theme *Managing challenges and catching opportunities for active learning in SRHR*.

### Theme: *Considering self and others when engaging with the uncomfortable dimensions of learning SRHR*

Through the interviews, it became clear that the participating students experienced learning about SRHR as different from learning about other subjects in the master’s program. As one student expressed it; “in SRHR every topic will be sensitive to someone.” The topics covered in the course were seen as both personal and culturally coded, leading to potentially uncomfortable situations in the learning environment. To actively participate in learning activities students therefore continuously balanced their own learning preferences and needs while being mindful of the others participating in the learning activities, captured in the overarching theme *Considering self and others when engaging with the uncomfortable dimensions of learning SRHR.*

## Discussion

In this study, we explored international students’ experiences of attending an international higher education course in SRHR in Sweden, focusing on active learning in relation to potentially sensitive topics. We found that active learning about sensitive SRHR topics relies on students and facilitators co-creation of an open, dynamic, and safe learning environment. The participants’ experiences and perceptions highlighted several dimensions of this process, including facilitators’ roles, facilitator-student hierarchies, pedagogical approaches, and student interactions.

### Facilitator strategies

Previous research highlights classroom discussions as central to support active learning [9, 15]. Findings from this study indicate that students’ willingness to participate is supported by facilitator strategies that manage proximity and distance when learning sensitive topics. These may include clarifying that disclosure of personal experiences should be avoided, emphasizing that learning activities aim at understanding diverse perspectives, encouraging an academic approach to discussions, and allowing students to regulate their level of engagement. These findings align with previous research on teaching controversial issues, which highlights the importance of pedagogical strategies that reduce feelings of personal exposure [6].

On the other hand, literature on teaching and learning sexuality in higher education, sometimes advocates personalization and self-reflection as means to highlight how perspectives, including student’s own, are shaped by sociocultural contexts and power relations [see for example: 1, 32]. Our findings suggest that such approaches require careful consideration and intentionality when addressing sensitive topics, as they may undermine trust and safety, and limit student engagement.

#### Co-creation - facilitators’ and students’ roles

Participants in this study emphasized the importance of facilitators in creating conditions for active learning. Participants described facilitators’ role in both course design and in classroom interactions, including tone of voice, choice of terminology, communication style, and responsiveness to feedback. Facilitators who invited student views, acknowledged ambiguity, and avoided overly direct correction were described as fostering a classroom climate characterized by trust, dialogue and mutual respect.

The students further described ways of engaging with sensitive topics that can be understood as face-saving strategies. According to Goffman, individuals seek to maintain a positive social self-image and employ strategies to protect their own and others’ “face” in social interactions [33]. Interviewees described several examples of face-saving strategies. For instance, choosing not to participate in discussions when holding minority views due to concerns about being misjudged, appearing disrespectful, or causing discomfort. Facilitators and peers also engaged in face-saving practices, such as reformulating or contextualizing potentially controversial statements. Trust that facilitators and peers would help co-create a respectful and face-maintaining environment contributed to students’ sense of emotional safety. This finding aligns with Zembylas’ [34] elaboration on the *pedagogy of discomfort* [35]. Zembylas’ relational perspective on emotions offers a useful lens to understand how feelings of discomfort or conversely, safety, emerge in SRHR classrooms through subtle dynamics of silence, active listening, and peer or facilitator responses. Accordingly, emotional environments are not just personal but co-created, and they shape the space for participation, disagreement, and ultimately, learning.

The findings also highlight tensions within dialogic pedagogies. While dialogue and face-saving practices can support student engagement with sensitive topics, students’ trust and sense of safety are undermined if facilitators fail to provide clear guidance or address disagreement, ambiguity, or inaccurate statements. A recent dissertation [36] supports this concern, illustrating that unmoderated so called *student discourse* may reproduce dominant student opinions rather than support subject-specific learning. Our findings further suggest that students’ trust in dialogic teaching is more fragile in guest-led sessions. This suggests that guest lecturers may need to be more explicit in guiding and clarifying discussions when using dialogic approaches.

The findings show that while trust is co-created, students ultimately expect facilitators to take responsibility for ensuring a safe learning environment. Our results indicate that facilitators are expected both to be subject-matter experts and to guide groups through challenging situations. This involves building and maintaining trust, role modelling constructive communication, and acting as guarantors of a safe learning environment.

### Disagreements in diverse classrooms and learning through discomfort

A related question concerns how assumed consensus shapes classroom climate and students’ sense of safety. Several students described discussions as more comfortable when they expected consensus, while disagreement, particularly around cultural, moral, or political values, was often emotionally challenging. However, the study found that emotional discomfort was not always a barrier to active learning. Instead, it sometimes prompted further reflection, information seeking, and discussion both in and outside the classroom, indicating that emotional resonance can deepen engagement with the subject matter. The results support previous studies showing that emotional intensity can be a necessary component of transformative learning [14]. The findings aligns with Illeris’s [37] understanding of emotion as one of three central dimensions of learning, where learning involves emotional aspects that contribute to integrating and internalizing experiences. Similarly, Zembyla’s [34] concept of “pedagogies of discomfort” propose that emotional disturbance can support transformative outcomes when carefully structured and facilitated.

Our findings echo research on teaching controversial issues [see for example: 5], which suggests that being challenged, alone, does not necessarily result in learning. Instead, learning occurs when pedagogical conditions are intentionally designed to support trust and engagement with difference [6]. In our material, being challenged was often experienced as both an intellectual and emotional process in which values, assumptions, or ways of knowing were questioned. In such situations, trust in the facilitator’s ability to manage both emotional intensity and the academic integrity of discussion was central. According to Glessmer et al. [38], trust is primarily cultivated through care, responsiveness, and attentiveness rather than subject matter expertise. Similarly, students in our study characterized trust as an affective process, fostered when they felt acknowledged, heard, and respected, even during disagreement or emotionally charged discussions.

Transformative learning may necessitate particular attention to the dual nature of the international classroom as both a challenge and a pedagogical asset. In our study, students came from diverse sociocultural contexts with varying norms concerning SRHR. This diversity made differences in values more visible. While students perceived the diversity to enrich learning, it was also perceived as challenging. Consistent with previous research on teaching sexuality in higher education [1, 32], our findings suggest that preparatory activities that address diverse values and perspectives help prepare students engage with the emotional and intellectual demands of learning SRHR. Importantly, while diversity can make heterogeneity more visible in international classrooms, facilitators and students may wrongfully assume homogeneity of values, opinions and knowledge concerning SRHR in less diverse settings.

Finally, our findings indicate that active learning can occur through experiences of discomfort, though its productivity depends on context and support. In our study, this discomfort can be understood as productive, leading to deepened learning, when it occurs in an environment characterized by trust that fosters collaborative engagement. Conversely, discomfort can also be productive when it leads to active learning in the form of individual, internal learning processes, where students reflect on peers’ comments yet without active participation in class, for example due to feelings of insecurity. Outside the classroom, discomfort sometimes spurred further reflection and reading at home, which contributed to learning but at an emotional and social cost in classroom. However, it is important to note also that discomfort may become a barrier to learning, potentially contributing to withdrawal, defensiveness or dropout when the perceived cost is too high, as discussed elsewhere [39, 40].

Taken together, the findings complicate a straightforward understanding of active learning as visible classroom participation, as the relationship between active participation and active learning appeared more complex. While trust and relational safety were described as prerequisites for participation, several students also reported consciously choosing to remain silent, withhold their opinions, or refrain from participating in order to avoid conflict or causing discomfort to others. This suggests a tension between the collective and individual dimensions of active learning. The conditions that contribute to a respectful and inclusive learning environment may simultaneously limit some students’ willingness to participate actively. Several students also described learning through listening, reflection, and continued processing of difficult issues outside the classroom. This raises questions about the relationship between active participation, active learning, and transformative learning when teaching addresses sensitive topics.

### Implications for educational practice

While our findings derive from one international SRHR course, they point towards pedagogical competences that may be relevant when teaching sensitive topics more broadly. Students consistently described trust as a prerequisite for engaging with discomfort in productive ways. This was supported through clear ground rules, opportunities to discuss issues on an academic rather than personal level, facilitators modelling of respectful dialogue and active facilitation of difficult discussions. These findings align with previous work highlighting the importance of structured approaches to controversial issues [6], relational aspects for trust building [38], and pedagogical practices that acknowledge rather than avoid emotional discomfort in learning [34]. Together the findings suggest that teaching sensitive topics requires competences that goes beyond subject expertise, including facilitation of dialogue, management of emotions, and the ability to support constructive engagement with disagreement. Such competences may therefore represent an important area for faculty development and facilitator training when teaching sensitive topics in higher education.

Future research should explore how facilitators themselves understand, develop and apply these competences in practice.

### Methodological considerations

A member check focus group was carried out by sharing preliminary result of the analysis with representatives from the study population. While member checks commonly are carried out with study participants [41], we chose to conduct an extended member check by involving also students who had not participated in the study. This was done to widen the perspectives on the analysis, given the limited size of the entire study population. The results from the member checks were integrated in the final analysis and contributed to the discussion of the manuscript.

The research team and authors of this article each contribute with important perspectives but also potential biases to the study. Three of the co-authors are all experienced teachers in SRHR and teach in the course that was being studied. The interviewers KB and CP give guest lectures in the course, and while this prior interaction with the participants might have strengthened the rapport it might also have influenced the respondents and what they chose to share in the interviews. TH is the course leader and main teacher of the course and has continued teaching the student cohort in other courses. And while this role offers a comprehensive understanding of the course content and dynamics, it is also likely to shape the interpretations of results. One of the co-authors (AP) is a former student of the course. Their previous experiences provided both valuable insights but might also have introduced personal biases due to their past participation in the course.

To enhance the readers’ understanding of the study’s transferability to other settings and groups, detailed descriptions of the study setting, study population, and the course were included in the article. Furthermore, a comprehensive description of the methods and an example of the analytical process were provided to enhance dependability and strengthen the overall trustworthiness [42] of the study. The findings should also be interpreted in relation to the Swedish higher education context as several participants contrasted this learning environment with more hierarchical educational experiences, suggesting that local expectations regarding teacher-student relationships and classroom participation may have influenced the findings.

## Conclusion

Our study supports and extends previous research on the pedagogical significance of emotional discomfort for learning and provides a detailed understanding of how students manage and react to affective tension in the classroom. Participants described an ongoing process of relational and ethical adjustment, choosing silence, modifying their language, or seeking additional knowledge outside the classroom. These findings indicate that the experienced discomfort has potential to be productive for transformative learning, given that the learning environment is curated by both facilitators and students into a safe environment characterized by trust, and the students perceived the establishment of ground rules for discussions as central to this. Our study also highlights the complex relationship between active participation, active learning, and transformative learning, and indicates possible new avenues for further research.

Through exploring the perspectives of the students when learning sensitive topics, our study spotlights specific expectations on the facilitator role and capacity that extends beyond the role of subject-matter expert. Future studies should further explore strategies and pedagogical choices employed by facilitators when teaching topics that could be considered sensitive and lead to discomfort, and the competences considered necessary to engage with such strategies and choices.

### Ethical statement

To protect the integrity of the participants from a comparatively small study population, representing just one student cohort (*N* = 41), information on countries of origin, ages and educational background of the participating students has been withheld. The study was conducted according to the guidelines of the Declaration of Helsinki and approved by the Swedish Ethical Approval Authority on September 17, 2024 [dnr. 2024-04738-01].

## Supplementary Information

Supplementary Material 1.

## Data Availability

Due to ethical concerns related to the risk of being able to identify individuals as a result of the small study population the full dataset cannot be shared publicly. De-identified data can be made available by the last author upon reasonable request.

## References

[1] Davis NJ. Taking Sex Seriously: Challenges in Teaching about Sexuality. Teach Sociol. 2005;33(1):16–31.

[2] Harding N. Reasonably uncomfortable: teaching sensitive material sensitively. In: Young S, Strudwick K, editors. Teaching criminology and criminal justice. Palgrave Macmillan, Cham: Springer International Publishing; 2022. pp. 199–224. 10.1007/978-3-031-14899-6_10.

[3] Pinar Alakoc B. Terror in the Classroom: Teaching Terrorism Without Terrorizing. J Political Sci Educ. 2019;15(2):218–36.

[4] Lowe P, Jones H. Teaching and Learning Sensitive Topics. Enhancing Learn Social Sci. 2010;2(3):1–7.

[5] Hess DE. Controversy in the classroom: the democratic power of discussion. 1st ed. Routledge; 2009.

[6] Kerr D, Huddleston T. Teaching controversial issues through education for democratic citizenship and human rights. Training pack for teachers. Council of Europe; 2020.

[7] Sanchez S, Venegas-Garcia C, Szott K, Gibbs L. Racial injustice, protests & reflexivity: student’s perspectives during COVID-19 class journaling activity. J Criminal Justice Educ. 2025:1–12. 10.1080/10511253.2025.2479672.

[8] Prince M. Does Active Learning Work? A Review of the Research. J Eng Educ. 2004;93(3):223–31.

[9] Doolittle P, Wojdak K, Walters A. Defining active learning: a restricted systematic review. Teach Learn Inq. 2023;11:1–24. 10.20343/teachlearninqu.11.25.

[10] Biggs J, Tang C. Teaching for quality learning at university – Fourth Edition. Croydon: Open University; 2011.

[11] Swedish Government. Högskoleförordning (1993:100). [The Swedish Higher Education Ordinance]. Utbildningsdepartementet [Ministry of education and research].

[12] Bickmore K, Parker C. Constructive Conflict Talk in Classrooms: Divergent Approaches to Addressing Divergent Perspectives. Theory Res Social Educ. 2014;42(3):291–335.

[13] Löfgren-Mårtenson L. Master i sexologi: pedagogiska resurser och utmaningar [Master in Sexology: Pedagogical resourses and challenges]. FoU-report 2008. Malmö University.

[14] Christie M, Carey M, Robertson A, Grainger P. Putting transformative learning theory into practice. Australian J Adult Learn. 2015;55(1):9–30.

[15] Padilla-Petry P, Rodríguez-Rodríguez J, Pérez-Hernando S. Overcoming the magnetism of lectures: teachers’ and students’ perceptions of and motivations regarding active learning in higher education. SAGE Open. 2025;15(1):1–13. 10.1177/21582440251323313.

[16] Nicol DJ, Boyle JT. Peer Instruction versus Class-wide Discussion in Large Classes: A comparison of two interaction methods in the wired classroom. Stud High Educ. 2003;28(4):457–73.

[17] Jagger S. Affective learning and the classroom debate. Innovations Educ Teach Int. 2013;50(1):38–50.

[18] Scriver S, Kennedy KM. Delivering education about sexual violence: reflections on the experience of teaching a sensitive topic in the social and health sciences. Ir Educational Stud. 2016;35(2):195–211.

[19] Parker-Shandal C. Participation in higher education classroom discussions: how students’ identities influence perspective taking and engagement. Teach Learn Inq. 2023;11.

[20] Hamilton A, Sechler A, Rothlisberger C, Smart S. et al. Student voices: discovering resistance. In: Tolman AO, Kremling J, editors. Why students resist learning: a practical model for understanding and helping students. 1st ed. Routledge; 2017. 10.4324/9781003448747-2.

[21] Fong M. Using technology to support discussions on sensitive topics in the study of business ethics. J Inform Technol Education: Res. 2015;14:243–56.

[22] States NE, Lovig C, Martin K, Nennig HT, Cole RS. Let students work: analysis of the role of differing facilitation on student engagement in a large stadium-style lecture hall. J Chem Educ. 2023;100(11):4237–48. 10.1021/acs.jchemed.3c00750 PMC10653220 38028750

[23] Cebula K, Macleod G, Stone K, Chan SWY. Student experiences of learning about potentially emotionally sensitive topics: trigger warnings are not the whole story. J Furth High Educ. 2022;46(8):1120–34.

[24] Heath M, Due C, Hamood W, Hutchinson AD, Leiman T, Maxfield K, Warland J. Teaching sensitive material: a multi-disciplinary perspective. Ergo. 2017;4(1):5–13.

[25] Rossouw J. Transforming Gender and Sexuality Education: An Autoethnographic Journey of Pedagogical Innovation in South African Higher Education. Social Sci. 2025;14(10):594.

[26] Svendby RB. Teaching sensitive and controversial issues in higher education: a discussion about teaching strategies by an ethics of care with sexually abused boys and men as the empirical example. Teach High Educ. 2025;30(5):1051–64.

[27] Malterud K, Siersma VD, Guassora AD. Sample Size in Qualitative Interview Studies: Guided by Information Power. Qual Health Res. 2016;26(13):1753–60. 10.1177/1049732315617444 26613970

[28] Graneheim UH, Lundman B. Qualitative content analysis in nursing research: concepts, procedures and measures to achieve trustworthiness. Nurse Educ Today. 2004;24(2):105–12. 10.1016/j.nedt.2003.10.001 14769454

[29] Graneheim UH, Lindgren BM, Lundman B. Methodological challenges in qualitative content analysis: A discussion paper. Nurse Educ Today. 2017;56:29–34. 10.1016/j.nedt.2017.06.002 28651100

[30] Lindgren BM, Lundman B, Graneheim UH. Abstraction and interpretation during the qualitative content analysis process. Int J Nurs Stud. 2020;108:103632. 10.1016/j.ijnurstu.2020.103632 32505813

[31] Lynn VA, Lennon-Dearing R. Putting people first: the power of inclusive language. J HIV/AIDS Social Serv. 2023;22(4):186–91.

[32] DeLamater J, Hyde JS, Allgeier ER. Teaching Human Sexuality: Personalizing the Impersonal Lecture. Teach Sociol. 1994;22(4):309–18.

[33] Goffman E. Interaction ritual: essays in face-to-face behavior. Routledge; 2017/1967.

[34] Zembylas M. Theory and methodology in researching emotions in education. Int J Res Method Educ. 2007;30(1):57–72.

[35] Boler M. Feeling power: emotions and education. New York, London: Routledge; 1999.

[36] Hildebrand LÄ. Från elevdiskurser till biologibruk. Sexualundervisning på gymnasiet. [From student discourses to biology - sex education in upper secondary education]. Doctoral dissertation. Department of Educational Sciences. Lund University. 2025.

[37] Illeris K. Towards a contemporary and comprehensive theory of learning. Int J Lifelong Educ. 2003;22(4):396–406.

[38] Glessmer MS, Persson P, Forsyth R. Engineering students trust teachers who ask, listen, and respond. Int J Acad Dev. 2025;30(1):106–19.

[39] Kumagai AK. Discomfort, Doubt, and the Edge of Learning. Acad Med. 2022;97(5):649–54. 10.1097/ACM.0000000000004588 35020611

[40] Selberg R. Trigger warnings – om undervisning och politisk gränshållning i 2020-talets sociala landskap. Högre utbildning. 2021;11(2):87–104.

[41] Birt L, Scott S, Cavers D, Campbell C, Walter F. Member checking: a tool to enhance trustworthiness or merely a nod to validation? Qual Health Res. 2016;26(13):1802–11. 10.1177/1049732316654870 27340178

[42] Amankwaa L. Creating protocols for trustworthiness in qualitative research. J Cult Divers. 2016;23(3):121–7. 29694754

