# Supplementary material for: Engaging with discomfort when learning sexual and reproductive health and rights. A qualitative content analysis of public health students’ experiences of active learning in an international master’s level course
Source: BMC Public Health. 2026 Sep 29;26:2702. doi: 10.1186/s12889-026-29680-4 (PMC13625496; doi:10.1186/s12889-026-29680-4)
Supplement: Supplementary file 1 [file 12889_2026_29680_MOESM1_ESM.pdf]

Version 1.0

Student Interview Guide

pedagogic research project

1. *Present yourself and the project. Give time to the student to read through the information letter.*

   Do you have any questions or anything you wonder about the project before we continue?

2. *Before starting the recording inform the student to keep the recording as anonymous as possible, this includes not mentioning names or being too detailed about other individuals. Emphasize the importance of understanding that participation is voluntary and that they can withdraw at any time or choose not to answer certain questions. Inform the student that you will start the recording.*

• Start recording

3. *Collect oral informed consent by using the consent form.*
   Structured questions: 1) Age, 2) Gender, 3) Region of origin, 4) Course

   Introductory question: What do you perceive as good teaching for your learning?
   Could you tell me about one specific time, from any course you ever had, when you felt especially comfortable participating in a classroom discussion or student discussion?
   Follow up: What made you feel especially comfortable at that time?

   Theme 1: What topics from classes did you consider more sensitive or difficult to discuss?
   Examples of follow-up questions: Why do you consider it sensitive/difficult? How did you experience learning about it? How did it make you feel? Why do you believe it made you feel that way? Did your feelings affect your willingness to participate in discussions?

   Theme 2: When learning about these subjects, how did the teacher influence the learning environment / your learning?
   Examples of follow-up questions: How do you view the teacher's responsibility? Can you describe a good/bad example of it? Can you describe methods or strategies that worked better/worse for your learning? What specific support measures or resources from the teacher have helped you feel safe to participate in the teaching?

   Theme 3: When learning about these subjects, how did your fellow students influence the learning environment?
   Examples of follow-up questions: How do you view the responsibility of the students? Can you describe any good/bad example of that? Are there ways this could have been be handled? How did the group dynamics in the classroom affect your participation?

   Theme 4: When learning about these subjects, what are the external factors (e.g. media, norms, etc.) influencing the learning environment?
   Examples of follow-up questions: How does this affect you and your learning? Why do you think this has an influence?

   Theme 5: Could you describe if there have been occasions where you felt you could not fully participate in discussions, not say what you wanted, or when you have been hesitant or felt uncomfortable to participate?
   Examples of follow-up questions: Why do you believe it made you feel that way? Did you share or discuss those experiences with any peer? What strategies did you use to handle the feelings of discomfort?

   Theme 6: Have there been occasions where you have been comfortable discussing one of the sensitive topics?
   Examples of follow-up questions: Why do you believe it made you feel that way? Did you share or discuss those experiences with any peer? What contributed to creating this environment?

   Ending: Is there something more you would like to share before we end, that you feel you have not had the oportunity to share during the interview?

■ Stop recording
